# Bacterial lysis or survival after infection with phage Sf14 depends on combined nutrient and temperature conditions

**DOI:** 10.1371/journal.pone.0319836

**Published:** 2025-03-25

**Authors:** Nykki D. Ross, Alexis L. Chin, Archana Pannuri, Sarah M. Doore

**Affiliations:** Department of Microbiology and Cell Science, University of Florida, Gainesville, Florida, United States of America; Kampala International University - Western Campus, UGANDA

## Abstract

Bacteriophage Sf14 is a Moogle-like myovirus that infects *Shigella flexneri*. *S. flexneri* is a human pathogen that replicates intracellularly in the intestine, but it persists in a low metabolic state in environmental fresh water sources. Though closely related to FelixO1, Moogleviruses were more recently discovered within the last 10 years; thus, mechanistic knowledge of their infection cycles is still being gathered. This work investigated the combined effects of temperature and nutrient concentration on both host growth and phage replication. In combination, a total of 16 different conditions were analyzed. Results indicate that nutrient-rich media facilitate shorter infection cycles and support phage production at all temperatures. As nutrient content decreased, temperature significantly affected both host cell replication and phage production. Results indicate phage genomes are entering the cells and genes are actively expressed; however, there is a significant delay in expression, which could allow bacterial populations to outpace phage growth.

## Introduction

Bacteriophages – also known as phages – are viruses that infect bacteria. They are ubiquitous in the environment and significantly outnumber their bacterial hosts [1–3]. Until recently, most identified and characterized phages infect *Escherichia coli* or *Salmonella enterica;* very little information was available on phages infecting *Shigella* species—specifically *Shigella flexneri*—despite their close relation to *E. coli* [4, 5]*. S. flexneri* is responsible for 164.7 million cases of bacillary dysentery worldwide each year; with 163.2 million cases in developing nations, it is the leading cause of shigellosis in these countries [6, 7]. Because of its small inoculum size and increasing resistance to antibiotics, phages infecting *S. flexneri* are being studied more in-depth to assess the possibility for use in phage therapy, environmental control, and detection [4,8,9].

Bacteriophage Sf14 is a Moogle-like myovirus that was isolated during a 2016 shigellosis outbreak in Michigan [10]. Unlike many previously described *S. flexneri* phages, it can infect multiple serotypes of *S. flexneri*. Sf14 and its 6 relatives isolated at this time all encode 25–27 tRNA genes and possess an uncommon genome size and capsid geometry. Prior to the discovery of these phages, genome sizes between 85.0 and 95.0 kbp were shared by only approximately 2% of all known phages, compared to ~ 35% of phage genomes between 35.0 and 45.0 kbp or ~ 16% of phages from 150.0 to 175.0 kbp [11, 12]. Similarly, only two previously identified phages shared its capsid geometry of T =  9 [13, 14], though this number has been recently increasing [15–19]. These features of the *S. flexneri* phage Sf14 make it an interesting target of research.

*Shigella* occupy at least two ecological niches: environmental surfacewater, where it persists in a dormant state; and the human gut, where it grows intracellularly in epithelial cells, resulting in disease. The full range of these conditions is not reflected in typical laboratory conditions, where phages are characterized and investigated for their suitability in application. To gain a better understanding of the growth kinetics of phage Sf14 and its host *S. flexneri*, we conducted a series of experiments in a variety of temperature and media conditions. The lab strain CFS100 – a nonvirulent derivative of model strain *S. flexneri* 2457T – was infected with Sf14 and grown under conditions simulating various environmental stressors. These included low-nutrient, low-temperature conditions (M9 media, 23°C) to mimic surfacewater; high-nutrient, high-temperature conditions (YT(2x) media, 37°C) to mimic a human cell; 42°C to induce heat stress; and all combinations between these ranges. Data from these experiments can be used to examine relationships between environmental conditions and growth phenotypes of both the host and phage.

## Results & discussion

### Nutrient-rich media supported phage production at all temperatures

**Fig 1:**
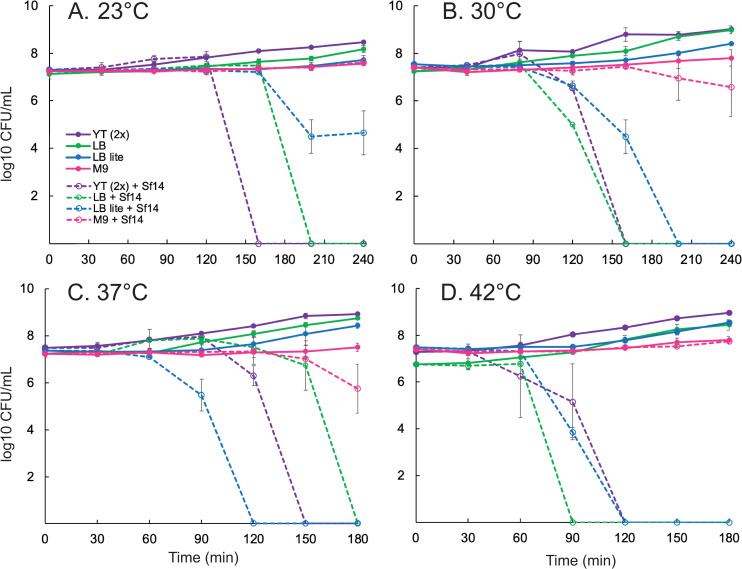
Growth of bacteria in various media and temperatures with or without Sf14 infection. Temperatures are A) 23°C, B) 30°C, C) 37°C, and D) 42°C. Different media types are indicated by color, with uninfected cultures represented by solid lines and phage-infected cultures represented by dashed lines. Numbers are reported as the log10 colony forming units (CFU) per mL. Temperatures are indicated in the upper left.

**Fig 2:**
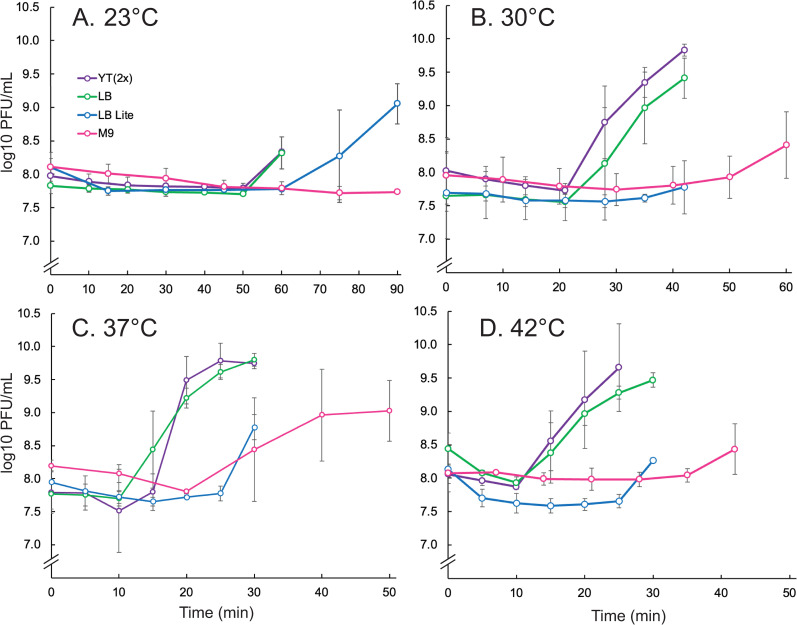
Growth of Sf14 in various media. Media type is indicated by line color, at temperatures A) 23°C, B) 30°C, C) 37°C, and D) 42°C. Numbers are reported as the log10 of plaque forming units (PFU) at the given time points.

The richest media tested here, a yeast-tryptone media at twice the typical concentration—designated YT(2x)—was routinely lysed at the low MOI in these experiments (Fig 1). Lysis was complete by about 150–200 min post infection regardless of temperature and the production of infectious particles exhibited a burst for all but room temperature. At room temperature, measured at approximately 23°C, particles increased modestly only towards the end of the timespan given for the assay (Fig 2A). Standard LB-Miller media was similar, suggesting the extra nutrient content had limited added benefit.

### Temperature significantly affected outcomes as nutrient content decreased

When nutrients from LB-Miller media were diluted 1:2—designated LB lite—the growth of bacteria was more variable. In general, bacteria grown in diluted LB grew more slowly and did not reach the same final concentration as in nutrient-rich media after 4 h. The lysis timing was also highly variable, with infected cultures in LB lite lysing later than LB at low temperatures or earlier than all cultures at high temperatures (Fig 1). The number of phage produced was also generally lower than phage produced in nutrient-rich cultures (Fig 2). These effects were most noticeable at temperatures of 30°C and above. At room temperature, infected cultures grown in LB lite did not fully lyse although the final PFU/mL reached similar levels compared to nutrient-rich media.

Growth of bacteria and phage in M9 minimal media was the slowest, as predicted, which was most likely due to the scarcest amount of nutrients. This effect was negligible compared to LB lite at 23°C and 30°C, likely due to overall slow growth of bacteria at these temperatures unless nutrients are abundant. Infected cultures grown in M9 did not lyse unless grown at 37°C. Despite this, phage were produced at all temperatures except 23°C at about a 10- to 100-fold decrease compared to cultures grown in YT(2x) or LB. Phage production was also generally delayed by 10–15 min compared to LB Lite, but final concentrations were equivalent between the two media types.

### Cultures did not lyse at either end of the temperature spectrum in nutrient-poor conditions

**Fig 3:**
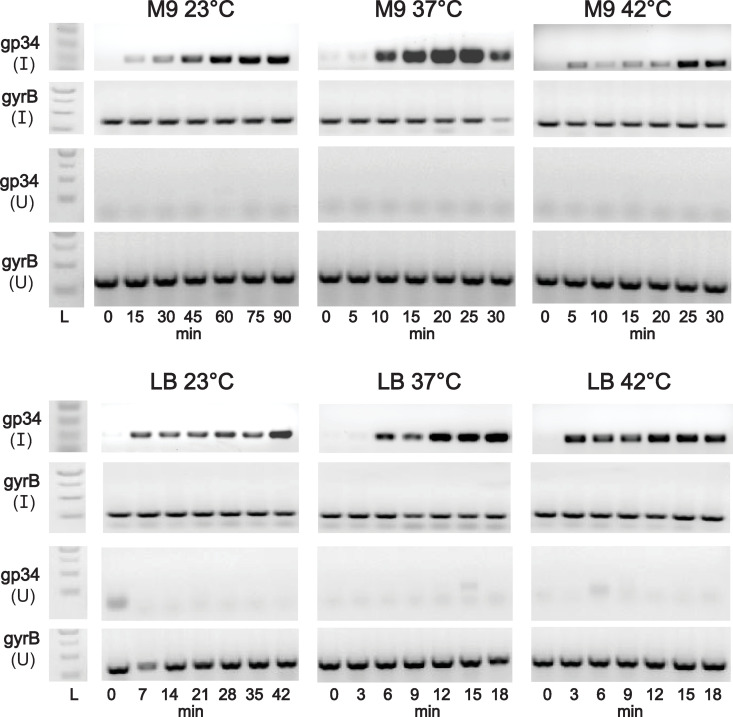
Expression of the major capsid protein *gp34* gene in nutrient-poor (M9) or -rich (LB) conditions at various temperatures. (I) corresponds to Sf14-infected samples, with (U) representing uninfected samples. The *gyrB* gene was included as a positive control to confirm presence and integrity of RNA from these samples. 30°C was excluded due to the similarity in phage replication kinetics at 30°C and 37°C.

Of interest is the pattern of growth in M9 at either end of the temperature spectrum. Perhaps not surprising due to poor phage production at 23°C, bacteria were able to grow consistently at this temperature despite phage infection. Whether this is due to lack of growth supporting productive infection or an overall equilibrium is unclear, though the phage late gene *gp34*, encoding the capsid protein, was produced at all temperatures (Fig 3). In M9 at 42°C, the number of PFU/mL increases 10-fold after about 40 minutes, but there is essentially no or minimal measurable difference between the growth of infected and uninfected cultures. Although it’s possible our phage production assay did not capture the true lysis timing, infected cells from the growth curves should not form colonies once they were removed from the culture and diluted for plating. Alternatively, the apparent lack of lysis could be due to a lower burst size in this condition, which may result in an undetectable impact on CFU concentration. Since our experiments were conducted at low MOI, these measurements may not be sensitive enough to detect low burst sizes.

Surviving bacteriophage predation under certain environmental conditions has also been seen in numerous other studies, including short- and long-term studies [20–26]. For example, thermal performance curves of *Pseudomonas fluorescens* were conducted in the presence and absence of its phage SBWφ2, with results demonstrating an increase in optimum growth temperature and a decrease in optimum growth rate when phage were present [20]. In studies on *E. coli* and its phage T4, temperature was shown to significantly impact both the cost of and the variation in developing phage resistance [21]. In our experimental setup, 42°C is likely above the optimum growth temperature for both phage and bacteria, but the short duration did not allow us to measure the cost of resistance, overcoming resistance, or subsequent changes in growth in this condition. When compounded with nutrient-poor media, growth of both phage and bacteria may have slowed to result in relatively stable dynamics.

Nutrients have also been examined as affecting growth dynamics and infection outcomes for *Salmonella* Enteriditis and vB_Sen_STGO-35-1 [22], *E. coli* and T4 [23–25], and *P. fluorescens* and SBWφ2 [26]; however, in these studies, host survival was more likely when nutrients were abundant, rather than when they were limited as shown in the present work. Some differences between our study and those referenced are the timescale of the experiments and the culturing methods. First, Barron-Montenegro et al. focused exclusively on nutrient-rich conditions during a 21-day course of experimental evolution [22]; second, Attrill et al. used microfluidics to examine single infections or small populations of fewer than ten cells per channel, while Bohannan and Lenski’s experiments were conducted over 12–25 days. In the final study by Gomez et al., plant-associated bacterium *P. fluorescens* and SBWφ2 dynamics were examined in the context of soil microcosms over 20 days [26]. The authors found that nutrient-rich conditions, bacterial density remained roughly consistent while phage density decreased to extinction. In populations without added nutrients, bacterial density declined while phage density stayed relatively consistent.

In comparing our methods to those of [22–26], spatial structure is heterogeneous in soil versus the liquid cultures used here, and it is likely that a longer-term culturing protocol for the nutrient-rich conditions we examined would allow for the emergence of resistance mutants. Resistance to predation can be costly, but the cost is surmountable in the resource-rich environments studied above. Additional resources can also increase the variation in predator-prey dynamics compared to relative stability in nutrient-poor conditions [24–25]. This stability in resource restricted conditions is consistent with our observations here, especially at non-optimal temperatures, even over a shorter time period. This further supports that a combination of nutrients, temperature, and host density or spatial structure will significantly affect infection outcomes.

## Conclusions

Bacteriophages have been studied for over a hundred years, but many experiments have been in standard nutrient-rich media at a fixed temperature that is favorable for bacterial growth. The experiments in this study illustrate the effect that nutrients and temperature can have on bacteriophage infection, and therefore on bacterial growth, in non-ideal conditions.

*S. flexneri* is a human pathogen, but it persists in the environment in a viable but non-culturable (VBNC) state. It appears to persist in fresh surface water, including sources of drinking water, from which it can emerge and cause disease outbreaks. Much of the research on *S. flexneri* is in nutrient-rich media at physiological temperatures, which is consistent with its environment as an enteric intracellular human pathogen. Less work has been done in conditions similar to freshwater, i.e., nutrient-poor media at environmental temperatures, from temperate water (~23°C) to tropical water (~30°C). Although 23°C is warmer than many temperate locations, it is still reflective of mean water temperature in a range of locations for at least part of the year [27].

Interestingly, *S. flexneri* phages are commonly found in freshwater even when *S. flexneri* or its molecular signatures are not readily isolated [10,28]. Results here suggest that phage can be produced at low levels in nutrient-poor media at 30°C but not 23°C, at least in the conditions measured. One possible reason is that phage cannot attach at low temperatures. Since this phenomenon was only observed in one type of media, it seems an unlikely explanation on its own. This effect was also seen in M9 at 42°C, where phages are eventually produced—albeit 25–35 min later than in other types of media. There could be a combination of factors contributing to poor phage production, e.g., low metabolic rate of bacteria combined with increased levels of lipopolysaccharide at low temperature [29]; or heat stress responses combined with altered levels of membrane proteins [30, 31]. To differentiate the effect of each factor, further studies are needed to address growth differences in these low nutrient conditions.

## Materials & methods

### Bacteria and phage strains and culture methods

*Shigella flexneri* strain CFS100 is an avirulent derivative of 2457T [32], lacking the virulence plasmid. Propagation and methods involving Sf14 has been described [10]. For all experiments, one LB plate was streaked to get isolated colonies of CFS100. A single colony of CFS100 was used to inoculate 2 mL of media overnight, as described below. The media for overnight incubation corresponded to the media used for growth kinetic analysis to avoid artifacts from switching abruptly between media and temperature conditions at the start of each experiment.

Four media conditions were used for bacterial growth and phage production assays. Standard LB broth was 25g/L Fisher Scientific LB Broth dry mix (10g/L casein protein, 5g/L yeast extract, 10g/L NaCl) dissolved in deionized water. The media YT(2x) was an approximate 2x concentration of yeast and protein compared to LB, containing 16g/L tryptone, 10g/L yeast extract, and 5g/L NaCl dissolved in diH_2_O to simulate nutrient-rich conditions. A half-concentration LB media – “LB lite” – simulated low nutrient conditions, containing 5g/L tryptone, 2.5g/L yeast extract, and 5g/L NaCl dissolved in diH_2_O. Finally, M9 Minimal media contains the minimum components needed for *S. flexneri* growth. The following components were added in order: 77.7mL sterile diH_2_O, 10 µ L 1M CaCl_2_, 20mL 5x M9 salts (5g/L NH_4_Cl, 15g/L KH_2_PO_4_, 2.5g/L NaCl, 30g/L Na_2_HPO_4_, diH_2_O), 500 µ L 20% glucose, 100 µ L 1M MgSO_4_, 200 µ L 100mM histidine, 600 µ L 100mM leucine, 300 µ L 100mM methionine, 400 µ L 25mM tryptophan, 30 µ L 1mM thiamine HCl, 100 µ L 100mM nicotinic acid (niacin). To measure phage production, 0.65% soft agar (1.6g nutrient broth, 1.0g NaCl, 1.3g agar, 200mL diH_2_O) was used in the double-layer plate assays.

To examine combined effects of culture conditions, experiments were performed at four different temperature conditions in each media type to simulate different environments. Standard laboratory conditions of 37°C in LB is both the optimal temperature for *S. flexneri* growth and the physiological temperature of the human host. To induce heat stress, cells were grown at 42°C. To mimic the environmental conditions where bacteriophage Sf14 is found, a temperature of 23°C and minimal M9 media was used. This is also a temperature at which *S. flexneri* is metabolically dormant in the environment. Finally, cells were grown at 30°C to determine the effects of reduced – but not minimal – temperature on bacterial growth and phage production. This resulted in a total of 16 culture conditions spanning all temperature and media combinations.

### *S. flexneri* growth kinetics

Two flasks were used for each temperature and media combination. An overnight culture of *S. flexneri* CFS100 was added to media at 1.6% inoculum for both flasks for YT(2x), LB, and LB lite; or 3.2% inoculum for M9. Flasks were grown at the indicated temperature while shaking until OD_600_ =  0.3 for YT(2x), LB, and LB lite; or OD_600_ =  0.1 for M9. Due to differences in cell size, these OD values correspond to approximately 1 x 10^8^ CFU/mL as determined in [33]. An aliquot was removed for counting CFU of the initial culture, then phage was added to one flask at an MOI of 0.1. Both flasks were incubated with shaking for 4–5 hours. Aliquots were removed every 40 minutes for immediate serial dilution in cold phage dilution buffer. Dilutions were plated on LB agar and incubated overnight at 37ºC, then colonies were counted the following morning to calculate CFU/mL. The experiment was complete either upon full lysis of the phage-infected culture or at the 5-hour mark.

### Sf14 growth kinetics

Bacteria were again grown as above in each temperature and media combination until OD_600_ =  0.3 or OD_600_ =  0.1. Phage was then added at an MOI of 0.5 to minimize multiply-infected cells and reduce potential lysis inhibition effects, but to increase signal for RT-PCR of phage genes as described below. The addition of phage was set as t = 0. At 0 and subsequent timepoints, 200 µ L of culture was removed and immediately diluted into cold buffer with lysozyme (1 mg/mL final concentration) and incubated on ice to stop bacterial growth and release intracellular virions. After 1 hr these were further diluted in 100-fold steps and plated for PFU using soft agar overlay. The soft agar contained 0.8% w/v Oxoid nutrient broth, 0.5% w/v NaCl, and 0.3% w/v agar. Plates were incubated overnight at 37°C and plaques were counted the following day to calculate PFU/mL.

### Sample collection for RNA extraction

Overnight cultures of CFS100 were grown as described. The following morning, 20mL cultures of CFS100 were prepared as above and upon reaching exponential phase of growth, phage Sf14 was added at an MOI of 0.5 to minimize effects of multiply-infected cells, including lysis inhibition. A second set did not have any phage added. Cells were harvested by pipetting 1mL aliquots into 1.5mL Eppendorf tubes containing 125 µ L cold phenol/EtOH stop solution (2mL water-saturated phenol and 38mL EtOH). Samples were centrifuged at 8,000 RPM for 2 minutes at 4ºC, the media was aspirated, and the remaining cell pellet was flash-frozen in liquid nitrogen. Samples were stored at ‒80°C until lysis and RNA extraction.

### RNA extraction

Cells were lysed by resuspending the pellet in 800 µ L of fresh 0.5mg/mL lysozyme solution and 80 µ L 10% SDS, then placed in a 64°C water bath for 1–2 minutes. In 2 mL microfuge tubes, samples were combined with an equal volume (1mL) of water-saturated phenol and inverted 10 times, then placed back in the 64°C water bath for 6 minutes, inverting 6–10 times every 40–60 seconds. Tubes chilled on ice and centrifuged at maximum speed for 10 minutes at 4°C. The aqueous layer was transferred to a fresh 2mL tube containing an equal volume of chloroform, inverted 6–10 times, and centrifuged at max speed (14,000 RPM) for 5 minutes at 4°C. The resulting aqueous layer was split equally into two 1.5mL tubes, to which 1/10 volume 3M NaOAc with 1mM EDTA (DEPC-treated) and 2–2.5 volumes of cold 100% EtOH were added. Samples were incubated at ‒80°C for at least 20 minutes up to overnight, then centrifuged at max speed for 25 minutes at 4°C. The resulting small white pellet was washed with 1mL 80% EtOH and centrifuged again at max speed for 5 minutes at 4°C. The ethanol was again carefully removed and pellets were allowed to air dry. Pellets were finally resuspended in 100 µ L RNase-free DEPC-treated H_2_O and the previously split samples were combined back into one tube, resulting in 200 µ L final volume.

### DNase I treatment of RNA samples

Each 200 µ L sample was treated with 0.5 µ L RiboLock RNase Inhibitor (Thermo Fisher), 50 µ L 5x DNase I Buffer (50mM MgCl2, 50mM Tris-HCl (pH 7.5), 5mM EDTA, 5mM DTT), 1 µ L RNase-free DNase (NEB), and incubated at 37°C for 30 minutes. For the first phenol extraction, an equal volume of water-saturated phenol was added to each sample tube and inverted 6–10 times, followed by centrifugation at > 10,000 RPM for 2–3 minutes at room temperature. After removing the top aqueous layer, an equal volume of 50:50 phenol/chloroform solution was added to each sample, then inverted 6–10 times and centrifuged using the same parameters. The top aqueous layer was removed and an equal volume of chloroform was added to each tube, inverted 6–10 times, and centrifuged as before. After removing the top aqueous layer, the chloroform extraction was repeated. Ethanol precipitation and washing the nucleic acid pellet was conducted as above. Air-dried pellets were resuspended in 50 µ L RNase-free H_2_O. The DNase I-treated RNA was quantified by spectrophotometry and stored at ‒80°C until use.

### Reverse transcription

To prepare samples for cDNA synthesis, the following was added to a tube for each RNA sample: 1 µ L 50 µ M random hexamers, 2 µ L 10 µ M dNTP mix, 1 µ L template RNA at 10 ng/ µ L, 13 µ L RNase- and DNase-free H_2_O. Samples were mixed and briefly centrifuged before incubation at 65ºC for 5 minutes to anneal primers. Following incubation, samples were moved immediately to ice and allowed to chill for at least 1 minute. RT master mix (4 µ L 5X Maxima H-Minus buffer, 100 µ L 100mM DTT, 1 µ L RiboLock RNase Inhibitor, 1 µ L Maxima H-Minus Reverse Transcriptase) was prepared, then 7 µ L of the mix was added to each sample containing annealed primers. Samples were incubated at 52°C for 10 minutes followed by incubation at 80ºC for 10 minutes. The thermocycler was allowed to cool to 10°C before removal of samples.

### PCR for Sf14 major capsid protein gp34 and bacterial gene *gyrB*

PCR was performed to determine timing of expression of the Sf14 major capsid protein *gp34*. Primers sequences were 5’ GAC CTG CGT ATG CAC ATG GAA GAT GAA GCT 3’ (forward) and 5’ GGT TCA TGT GAG CCT GAA CAC CGT CAG TAC 3’ (reverse). PCR was also performed for uninfected cells using the Sf14 *gp34* gene, plus the CFS100 housekeeping gene *gyrB* to confirm that RNA was successfully extracted and reverse transcribed. Primer sequences for *gyrB* were 5’ GTG GAA GTG GTC TTC TTT GCC GTC ACG 3’ (forward) and 5’ CGT TGG TGT TTC GGT AGT AAA CGC CC 3’ (reverse). A master mix was prepared containing 29 µ L RNase- and DNase-free H_2_O, 10 µ L 5X Taq buffer (NEB), 4 µ L 2.5mM dNTPs, 2.5 µ L each forward and reverse primer, and 1 µ L Taq polymerase (NEB). 49 µ L of this mix was distributed to 0.2mL tubes containing 1 µ L cDNA. Samples were incubated in the thermocycler following the standard Taq parameters and run on a 1% agarose gel. Bands were imaged using the iBright imaging system.

## Supporting information

S1 Fig Growth of bacteria in various media and temperatures with or without Sf14 infection. Graphs of data shown in Fig 1 but grouped by media type rather than temperature. Media type is indicated at the top of each graph, with different temperatures indicated by color. Uninfected cultures are represented by solid lines and phage-infected cultures are represented by dashed lines. Numbers are reported as the log10 colony forming units (CFU) per mL.
(TIF)

S2 Fig Growth of Sf14 in various media and temperatures. Graphs of data shown in Fig 2 but grouped by media type rather than temperature. Media type is indicated at the top of each graph, with different temperatures indicated by color. Numbers are reported as the log10 of plaque forming units (PFU) at the given time points.
(TIF)

S1 File CFU counts and calculations.
Data from all replicates for bacterial growth curves, grouped by temperature or by media type.
(XLSX)

S2 File PFU counts and calculations.
Data from all replicates for phage growth curves, grouped by temperature or by media type.
(XLSX)

S3 File Original, uncropped gel images.
All original images of DNA gels used in Fig 3.
(PDF)
