# Supplementary figures and images for: Bacterial lysis or survival after infection with phage Sf14 depends on combined nutrient and temperature conditions
Source: PLoS One. 2025 Mar 25;20(3):e0319836. doi: 10.1371/journal.pone.0319836 (PMC11936213; doi:10.1371/journal.pone.0319836)

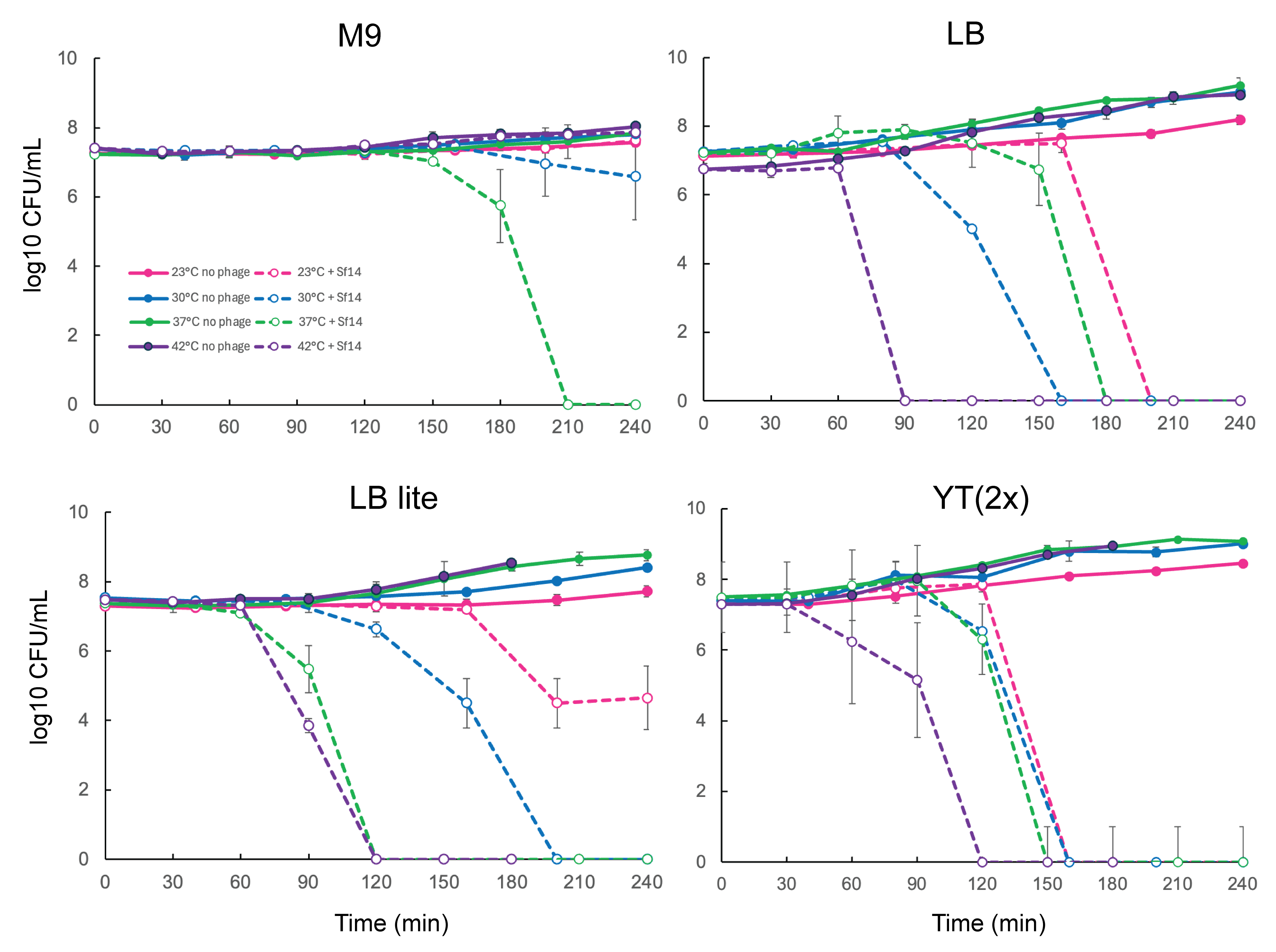

Supplement: S1 Fig — Graphs of data shown in Fig 1 but grouped by media type rather than temperature. Media type is indicated at the top of each graph, with different temperatures indicated by color. Uninfected cultures are represented by solid lines and phage-infected cultures are represented by dashed lines. Numbers are reported as the log10 colony forming units (CFU) per mL. (TIF) [file pone.0319836.s001.tif]

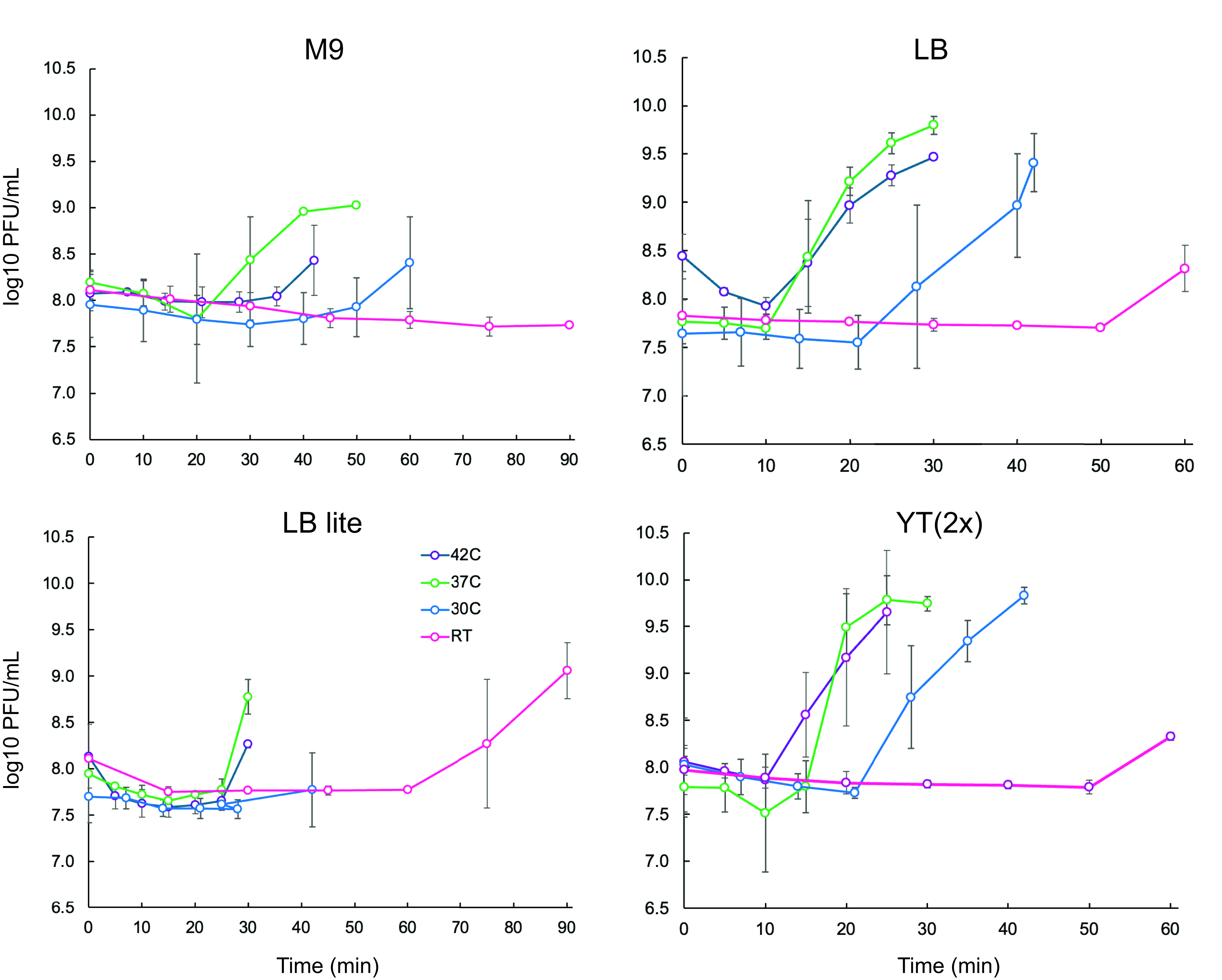

Supplement: S2 Fig — Graphs of data shown in Fig 2 but grouped by media type rather than temperature. Media type is indicated at the top of each graph, with different temperatures indicated by color. Numbers are reported as the log10 of plaque forming units (PFU) at the given time points. (TIF) [file pone.0319836.s002.tif]

Infected

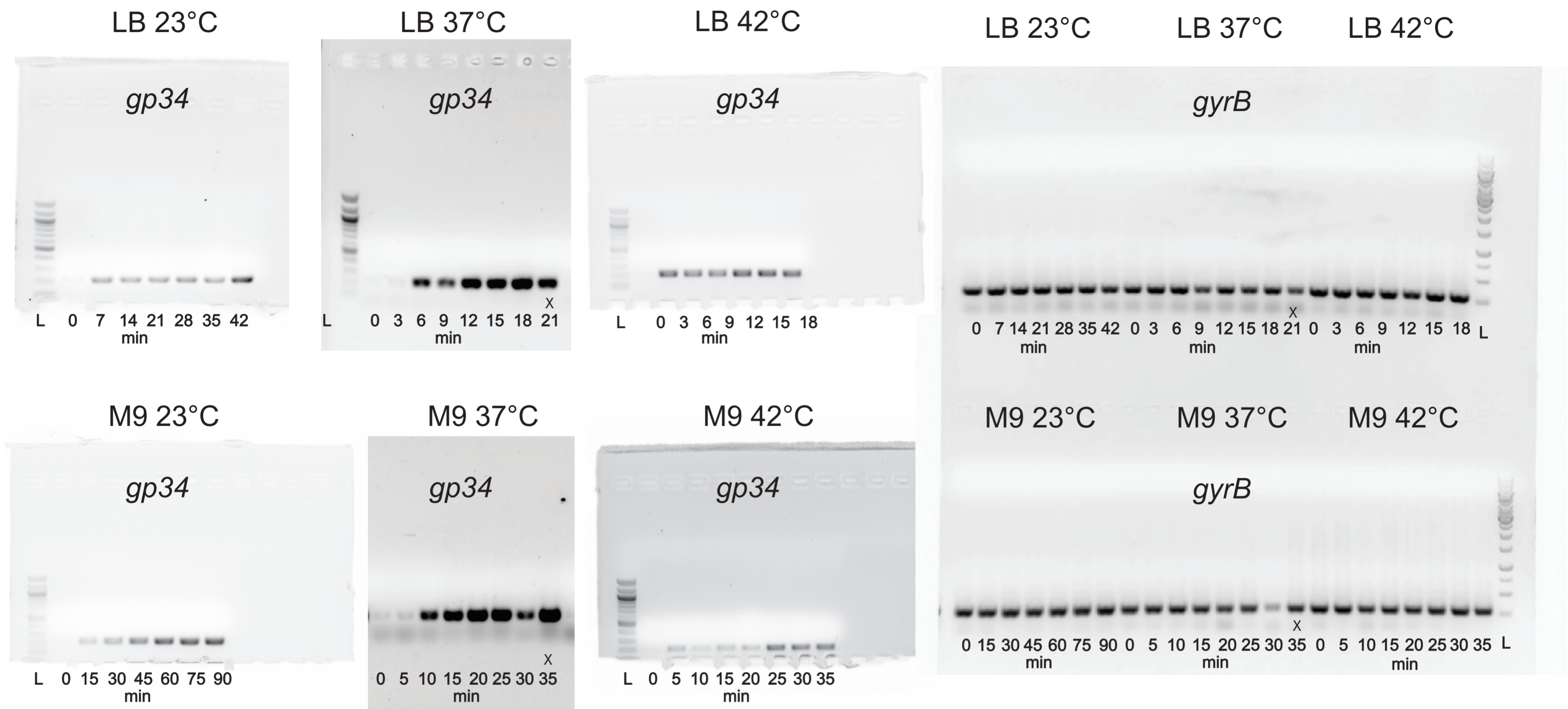

Uninfected

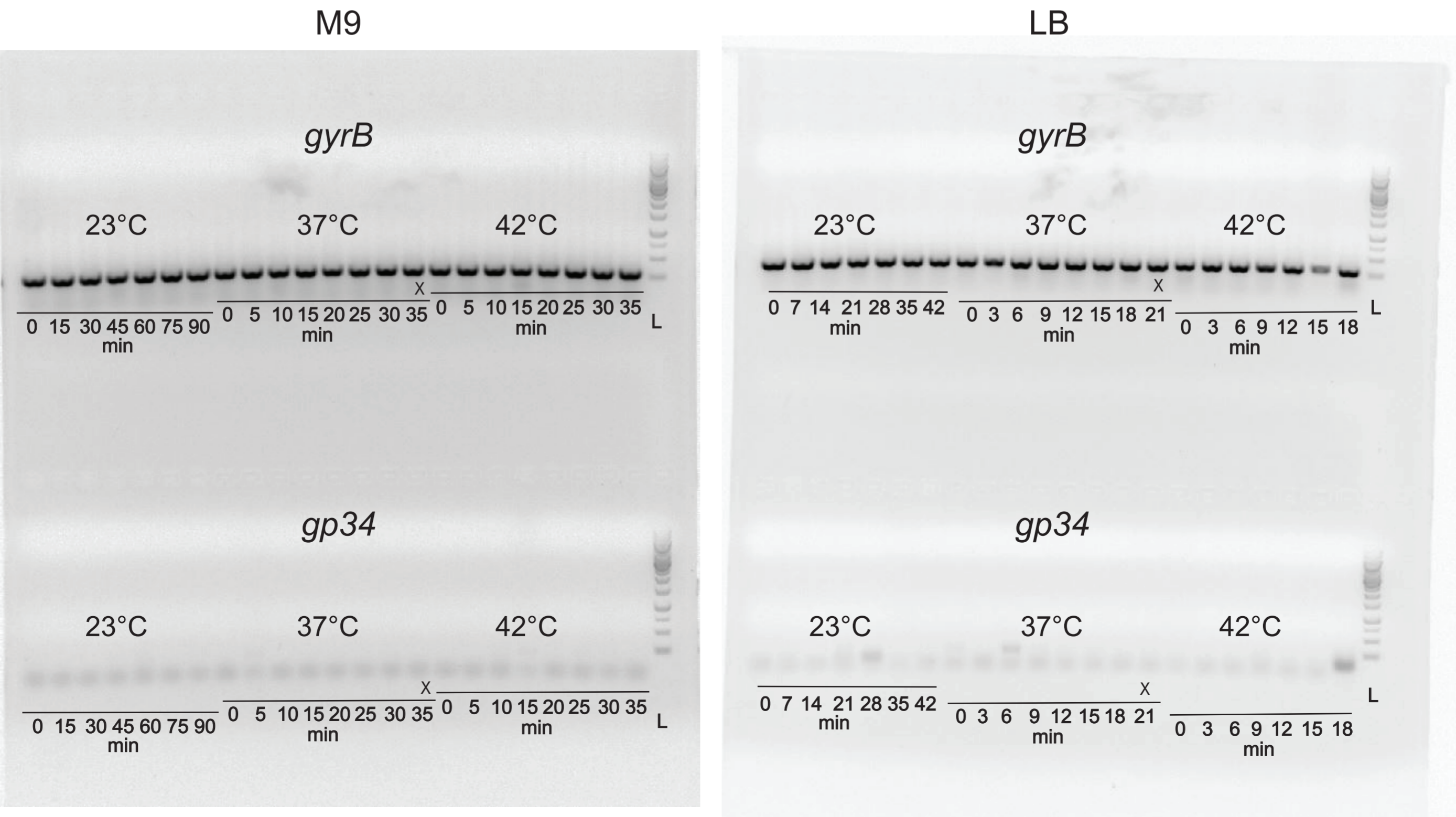

Supplement: S3 File — All original images of DNA gels used in Fig 3. (PDF) [file pone.0319836.s005.pdf]
